# Neural Isolation of the Olfactory Bulbs Severely Impairs Taste-Guided Behavior to Normally Preferred, But Not Avoided, Stimuli

**DOI:** 10.1523/ENEURO.0026-20.2020

**Published:** 2020-03-23

**Authors:** Chizuko Inui-Yamamoto, Ginger D. Blonde, Fabienne Schmid, Lauren Mariotti, Matias Campora, Tadashi Inui, Lindsey A. Schier, Alan C. Spector

**Affiliations:** 1 Department of Psychology and Program in Neuroscience, Florida State University, Tallahassee, Florida 32306; 2 Department of Oral Anatomy and Developmental Biology, Osaka University Graduate School of Dentistry, Osaka 565-0871, Japan; 3 Department of Biological Sciences, University of Southern California, Los Angeles, California 90089

**Keywords:** affect, gustatory system, olfactory system, reward, smell, taste

## Abstract

Here we systematically tested the hypothesis that motivated behavioral responsiveness to preferred and avoided taste compounds is relatively independent of the olfactory system in mice whose olfactory bulbs (main and accessory) were surgically disconnected from the rest of the brain [bulbotomy (BULBx)]. BULBx was confirmed histologically as well as functionally with the buried food test. In brief access taste tests, animals received 10-s trials of various concentrations of a taste compound delivered quasirandomly. BULBx C57BL/6 (B6) mice displayed severely blunted concentration-dependent licking for the disaccharide sucrose, the maltodextrin Maltrin, and the fat emulsion Intralipid relative to their sham-operated controls (SHAM B6). Licking for the noncaloric sweetener saccharin was also blunted by bulbotomy, but less so. As expected, mice lacking a functional “sweet” receptor [T1R2+T1R3 knockout (KO)] displayed concentration-dependent responsiveness to Maltrin and severely attenuated licking to sucrose. Like in B6 mice, responsiveness to both stimuli was exceptionally curtailed by bulbotomy. In contrast to these deficits in taste-guided behavior for unconditionally preferred stimuli, BULBx in B6 and KO mice did not alter concentration-dependent decreases for the representative avoided stimuli quinine and citric acid. Nor did it temper the intake of and preference for high concentrations of affectively positive stimuli when presented in long-term (23-h) two-bottle tests, demonstrating that the surgery does not lead to a generalized motivational deficit. Collectively, these behavioral results demonstrate that specific aspects of taste-guided ingestive motivation are profoundly disturbed by eliminating the anatomic connections between the main/accessory olfactory bulbs and the rest of the brain.

## Significance Statement

The senses of taste and smell have long been known to interact to contribute to the perception of flavor, but they are generally thought to operate independently. Here we found that severing the anatomic connections between the olfactory bulb and the rest of the brain severely blunts the taste-motivated licking responses of mice to brief presentations of a wide range of concentrations of highly preferred stimuli that have an unconditioned positive affective valence. In contrast, the concentration-dependent decrease in the licking of taste stimuli considered aversive was impervious to this neural transection. These results underscore the importance of understanding gustatory function in a broader context that involves a significant contribution from the olfactory/vomeronasal system in the maintenance of certain taste-motivated behaviors.

## Introduction

Historically, research on the senses of taste and smell, for the most part, has been pursued independently. Nevertheless, it is well understood that humans integrate sensations from the olfactory, gustatory, and trigeminal sensory systems to form what is perceptually referred to as flavor (Small and Prescott, 2005). Indeed, there are psychophysical and functional brain imaging data in humans that suggest that the olfactory and gustatory systems interact (Dalton et al., 2000; Small et al., 2004; Small and Prescott, 2005; Bartoshuk and Klee, 2013). In rodent models, the responsiveness of some neurons in the central gustatory system can be modulated by olfactory stimulation and vice versa (Van Buskirk and Erickson, 1977; Escanilla et al., 2015; Maier et al., 2015; Maier, 2017; Samuelsen and Fontanini, 2017).

Although influenced by olfactory stimulation, taste responsiveness is generally not considered to be dependent on it. For example, the chorda tympani response to lingual application of sucrose, which is known to activate the T1R2+T1R3 heterodimer expressed in a subpopulation of taste bud cells (Nelson et al., 2001; Zhang et al., 2003), in mice that were rendered anosmic by irrigation of the nasal epithelium with zinc sulfate is normal, suggesting that the hindbrain is receiving the signal generated by the activation of taste bud cells by the sugar (Uebayashi et al., 2001). Decerebrate rats that have their forebrain, including any descending projections from the olfactory system, neurally disconnected from the rest of the nervous system, display relatively normal reflex-like oromotor and somatic responses to basic taste stimuli (Grill and Norgren, 1978). Nevertheless, how peripheral taste signals are processed by central gustatory circuits in animals with disruption of the olfactory system has not been systematically examined. Moreover, there are some studies that have shown that interruption of olfactory function, either through zinc sulfate treatment of the epithelium or damage to the olfactory bulb, leads to altered intake and a preference for certain taste compounds (Vigorito and Sclafani, 1987; Ramirez, 1993; Lien et al., 1999; Takeda et al., 2001; Chambliss et al., 2004; Romeas et al., 2009; Zukerman et al., 2009a; Sato et al., 2010). This literature, however, is uneven with respect to the severity of the effects and the taste stimuli that are implicated.

One interpretive limitation regarding the effects of manipulations of the olfactory system on the intake of and preference for taste stimuli is that these tests are vulnerable to the influence of nongustatory factors such as postingestive events. The shortcomings of intake tests can be largely circumvented by the application of a behavioral procedure referred to as the brief access test (Young and Trafton, 1964; Davis, 1973; Smith, 2001; Glendinning et al., 2002; Spector, 2003). As its name implies, this method involves the pseudorandom presentation of very short duration (i.e., a few seconds) trials of various taste stimuli to the animal and the quantification of the licking response. The fact that the brief access test involves the measurement of an immediate licking response to a very small volume of stimulus increases confidence that the behavior is guided by orosensory signals. These experimental virtues confer distinct advantages in assessments of the hedonic evaluation of a taste stimulus in an animal compared with intake and preference tests.

The application of this method to examine the contribution of the olfactory system to taste-guided behavior has been, so far, limited (Uebayashi et al., 2001). In a recent study, we found that bulbotomy (BULBx), the surgical interruption of the connections between the olfactory bulb and more central brain sites, diminished the ability of mice to express an experience-dependent affective discrimination between glucose and fructose in a brief access test (Schier et al., 2019). In fact, the bulbotomized animals expressed blunted licking responses to both sugars. Here, in an effort to more systematically examine whether the neural isolation of the olfactory bulbs impairs the motivational salience of taste stimuli, we tested the concentration-dependent licking responses of bulbotomized and sham-operated mice to a variety of preferred and nonpreferred chemical solutions delivered in a brief access test. We also tested these mice for their intake of and preference for maltodextrin (i.e., Maltrin) and sucrose across a range of concentrations.

## Materials and Methods

### Subjects

In all experiments outlined below, C57BL/6J mice (B6; The Jackson Laboratory; age range, 10–14 weeks at the start of the study) were used. Separate mice were used for each experiment. Knock-out (KO) mice lacking both components of the heterodimer T1R2+T1R3 receptor and 20–33 weeks old at the start of study were also used in experiments 1 and 2. These mice were generated from initial breeding pairs that were null for the *Tas1r2* or *Tas1r3* gene (derived from 129X1/SvJ mice backcrossed with B6 mice, and generously provided by Charles Zuker, then at the University of California, San Diego). Homozygous null mice for either gene were paired together to generate mice heterozygous for *Tas1r2* and *Tas1r3*. These mice were then paired so that later generations would include mice homozygous null for both genes. At the end of study, KO mouse genotypes were confirmed independently by Transnetyx via quantitative PCR. A single nucleotide polymorphism genome analysis of known polymorphisms between B6 and 129X1/SvJ mice (completed by The Jackson Laboratory) showed that the KO mice have an ∼20–30% contribution from the 129X1/SvJ strain. Throughout all experiments, mice were single housed in polycarbonate mouse cages in a room with computer-controlled temperature, humidity, and lights (12 h light/dark cycle). A cotton-fiber nestlet (Ancare) was provided for environmental enrichment. Mice were provided *ad libitum* chow (Rodent Laboratory Chow 5001, Nestlé Purina Petcare) and reverse osmosis-deionized water, except where noted otherwise below. While under food or water restriction, body mass and body condition scores were measured daily (Ullman-Culleré and Foltz, 1999). Any animal falling to <80–85% of its *ad libitum* body mass or with a decrease in body condition score was given 1–2 ml of supplemental water after the end of the daily session. All procedures were approved by the Animal Care and Use Committee at the Florida State University.

### Taste stimuli

Taste solutions were prepared daily from reagent grade chemicals mixed in reverse osmosis-deionized water. Stimuli consisted of sucrose (Macron Fine Chemicals), Maltrin-580 (Grain Processing Corporation), citric acid, quinine hydrochloride, Intralipid, and sodium saccharin (Sigma-Aldrich).

### Apparatus

Brief access training and testing took place in a computer-controlled gustometer, described in detail previously (Spector et al., 2015). Briefly, a mouse was placed in a rectangular testing chamber consisting of three Plexiglas sides and a stainless steel front panel. A central slot allowed access to fluid deposited on a borosilicate sample ball that rotates around a horizontal axis. When the mouse licked, a force transducer connected to the ball would register the lick; fluid was deposited on the ball via tubing connected to a syringe that was mounted to a stepper motor calibrated to deliver a precise volume (∼1 μl/lick). An isolated syringe and tubing were used to deliver each stimulus concentration. Tubing was threaded through a circular motorized turret that would rotate to position the appropriate tubing in place to deliver the sample for each trial. Between trials, the sample ball was rotated away from the access slot, whereupon it was rinsed with reverse osmosis-deionized water and dried with pressurized air before being repositioned to receive the next stimulus. The testing chamber was housed within a sound attenuation chamber during all sessions. Masking noise was presented to reduce auditory cues. Air was drawn away from the sample ball to attenuate potential olfactory cues, via ductwork connected to an exhaust fan. A stainless steel shield was placed in front of the turret to preclude visual cues.

### Bulbotomy

For BULBx surgeries, mice were anesthetized with isoflurane (induction, 5%; maintenance, 1–5%) and placed in a stereotaxic device with nonpuncture earbars. The aseptic technique was followed to prepare a midline incision in the scalp, exposing the skull overlying the olfactory bulbs. A small (1-to 2-mm-diameter) burr hole was drilled into the skull on either side of midline. The overlying dura was carefully cut and retracted, exposing the dorsal surface of the bulbs and the rostral forebrain. Using a blunt sterile 23 ga needle, the tissue between the bulbs and forebrain were gently transected with the aid of a surgical microscope to minimize damage to vasculature and forebrain tissue. A sterile hemostatic gelatin sponge was placed in the cavity to minimize bleeding. The incision was closed with a 5–0 silk suture. For sham surgeries, the bulbs and rostral forebrain were exposed but no transections were made. On the day of surgery, mice were given a single dose of buprenorphine (0.1 mg/kg, s.c.). On the day of and the day after surgery, mice were given prophylactic antibiotics (gentamicin, 8 mg/kg, s.c.) and analgesics (carprofen, 5 mg/kg, s.c.). Food and water intake and body weight were monitored daily during recovery. Mice were allowed 10–29 d of recovery, depending on the experiment, before food or fluid restriction was required for postsurgical brief access testing (see below).

### Buried food test

Before surgery (in experiments 1–3) and again after the end of postsurgical testing, all mice were given a buried food test (BFT) to assess olfactory function using 0.5 g of a potato crisp. Mice were given a 0.5 g sample of potato crisp (Pringles Original, Kellogg Company) at least 1 d before the test to allow acclimation to the new food. Chow was removed from the home cage ∼22 h before the test. On the test day, mice were first placed in a clean standard mouse housing cage for 10 min to habituate. After 10 min, the mouse was moved to the center of the test cage, which was a standard mouse polycarbonate cage containing a 0.5 g crisp buried under 2 cm of bedding in a randomized quadrant. Latency for the mouse to find and engage with (i.e., pick it up or bite it) the crisp was measured. The mouse was immediately returned to its home cage without allowing it to consume the crisp. If a mouse failed to locate the crisp within 15 min, the test was terminated and the mouse was given a visual food test to confirm that the animal was motivated to engage the crisp. Here, the mouse was placed in the center of another clean cage containing a 0.5 g crisp placed on the surface of the bedding. Latency to find and engage the crisp was measured, with a maximum time allowed of 15 min. The mouse was immediately returned to the home cage without consuming the crisp.

### Histology

Following the postsurgical BFT, all mice were injected with an overdose of a euthanasia solution (0.1 ml; Euthasol) and transcardially perfused with heparinized saline and 4% paraformaldehyde. Observations of any connections between olfactory bulbs and the forebrain were noted. Brains were postfixed in the skull, with the dorsal bone removed, in 4% paraformaldehyde for at least 2 d and then completely removed from the skull and immersed in 20% sucrose in PBS. Tissue was then sliced frozen on a cryostat in 60 μm sagittal sections, mounted onto chrome-alum subbed slides, and stained with thionin. Sections were visualized under a light microscope and checked for the plane and extent of transection by an observer unaware of the surgical group, genotype, or behavioral outcomes of the mouse.

### Experimental design and statistical analysis

#### Brief access test

As noted above, the brief access test is well suited to assess affective responsiveness in rodents to taste stimuli. Rats and mice will increase their licking of sugars, fat emulsions, and maltodextrin solutions, as concentration is raised when tested in a fasted state (to promote stimulus sampling) or even when tested nondeprived. It is equally the case that water-deprived rodents will decrease their licking of certain qualitative classes of taste stimuli, including bitter ligands and acids, in a concentration-dependent manner. It is well documented that a variety of manipulations of the peripheral and central gustatory system, including genetic, pharmacological, anatomic, and optogenetic manipulations, can markedly modulate this behavior (Yamamoto and Asai, 1986; Krimm et al., 1987; Spector et al., 1993; St John et al., 1994; Zhang et al., 2003; Mathes and Spector, 2011; Treesukosol et al., 2011b; Wang et al., 2018). Accordingly, we chose this procedure as the principal assay of taste-guided motivation to lick in SHAM and BULBx mice.

During presurgical brief access training for all experiments, mice were water restricted for ∼23.5 h with fluid being provided during the 30-min sessions. For 2–3 d, free access to water was provided in a single 30-min trial. Then for 3 d, mice were given water in trials. For these and all test sessions, a 10-s trial began when the mouse licked the sample ball twice within 250 ms and a ∼10 μl preload of the stimulus was deposited on the sample ball. Mice could initiate as many trials as possible during the session and lick as much as possible within each trial.

In experiment 1, training concluded with the water-restricted mice receiving a session with a concentration series of Maltrin (1–32%, w/v) and water randomly presented (without replacement) in blocks of seven trials. At the end of training, water bottles were returned, and the animals were given ∼48 h to rehydrate. All testing for this experiment occurred under a food-restricted state. Food was removed from the home cage ∼23.5 h before each test, and returned ∼1 h after the end of the test session. Restriction did not occur again for 24–48 h. The water and food restriction schedules were used to promote stimulus sampling. Each stimulus was tested for three sessions. Mice were tested presurgically with Maltrin, and postsurgically with Maltrin and then sucrose (1–32% w/v and water).

In experiment 2, training concluded after the water training described above. There was no presurgical testing. Postsurgical testing was conducted under water restriction, with water bottles removed before the first session for the week, and testing conducted on consecutive days. The first 2 d of each week were all water trials, followed by 3 d of sessions with either citric acid (0.1–100 mm) or quinine (0.01–20 mm) presented randomly (without replacement) along with water in blocks of seven trials. To encourage licking and to help rinse away the stimulus, 1 s water access (maximum of 5 licks) was offered before each taste trial. Approximately half of the mice from each group were given citric acid in the first week, and the others were tested with quinine. Water bottles were returned for ∼48 h and removed again ∼22 h before the second week of testing, during which mice were presented with water trials for 2 d, and then 3 d with the second stimulus.

In experiment 3, training concluded with the water-restricted mice receiving a session including water and a concentration series of Intralipid (0.625–20% v/v) presented in blocks of seven trials randomized without replacement. After water bottles were replaced, all testing was conducted as described for experiment 1. Presurgical testing was performed using the Intralipid concentration series. Postsurgical testing used Intralipid, sucrose (1–32% w/v), and then Na-saccharin (0.1–50 mM).

After the last day of testing for all experiments, food (experiments 1 and 3) or water (experiment 2) was returned. After 5 d, mice were given the postsurgical buried food test. In experiment 1, 16 B6 mice (8 male, 8 female) and 14 KO mice (10 male, 4 female) underwent BULBx. Twelve B6 mice (6 male, 6 female) and 12 KO mice (8 male, 4 female) underwent SHAM surgery. In experiment 2, 16 B6 mice (8 male, 8 female) and 15 KO mice (8 male, 7 female) underwent BULBx. Twelve B6 mice (6 male, 6 female) and 13 KO mice (6 male, 7 female) underwent SHAM surgery. In experiment 3, 20 B6 mice underwent BULBx (10 male, 10 female). Twelve B6 mice underwent SHAM surgery (6 male, 6 female). The surgical group assignments were made to match the groups as closely as possible on body weight and presurgical licking behavior and to distribute the available males and females. Two mice (1 B6, 1 KO) from experiment 1, seven mice from experiment 2 (4 B6, 3 KO), and one mouse from experiment 3 died from surgical complications reducing the sample sizes above accordingly.

#### Postsurgical 23 h two-bottle preference test

In experiment 4, 16 B6 mice (8 male, 8 female) and 8 KO mice (4 male, 4 female) underwent BULBx; 8 B6 mice (4 male, 4 female) and 8 KO mice (4 male, 4 female) underwent SHAM surgery. The surgical group assignments were made to match the groups as closely as possible on body weight and to distribute the available males and females. Four mice (3 B6, 1 KO) died from surgical complications, reducing the sample size accordingly. After recovery, intake and preference for multiple concentrations of sucrose and Maltrin (2%, 8%, 16%, and 32% w/v) were assessed postsurgically in a series of two-bottle tests. A single concentration of stimulus and water was presented concurrently in 23-h tests. Weights for each bottle, and food, were measured at the beginning and end of each test period. Body mass was measured at the beginning of each test. The experiment started with presenting animals with two bottles of water for two 23-h periods. Then the solutions were presented in an ascending concentration series, with the isocaloric concentrations of each tastant tested sequentially. Each stimulus was presented in two 23-h periods, followed immediately by the isocaloric concentration of the second stimulus, also tested for two 23-h periods. Stimulus presentation was counterbalanced across groups, with half of the animals starting with sucrose and the other half starting with Maltrin. Between each 23-h test day, bottles were rinsed and refilled with fresh stimulus, and the position on the cage was rotated. There was a 72-h separation between concentrations.

#### Data analysis

Licks taken in each brief access trial were recorded by computer. On the first trial of a session, if a mouse did not approach the sample ball within ∼20 s, the sample ball was cleaned and the next trial presented, and this activity generally provoked stimulus sampling. In these cases, the first trial was not included in the analysis. Data were combined across the three test sessions for each stimulus. For Maltrin, sucrose, Intralipid, and saccharin, taste trial data were standardized across animals by subtracting the average licks to water from the average licks for each concentration for each animal (taste − water licks). For citric acid and quinine, data were standardized by dividing the average licks for each concentration by the average lick to water for each animal (taste/water ratio). These two methods adjust for individual differences in baseline lick rates and have been shown to be sensitive to manipulations of the gustatory system in rats and mice (St John et al., 1994; Spector, 2003; Dotson et al., 2005; Treesukosol et al., 2009). The reason the taste/water ratio is not used for normally preferred stimuli is because lick rates for water are often times quite low and thus minor random variation in the denominator across animals and sessions can have an inappropriately large impact on the value of the ratio. Group averages (±SE) were calculated for each stimulus. In the graphs, nonlinear regression (Eq. 1) was used when possible to fit the mean concentration–response functions to sigmoidal logistic function of the basic form, as follows:

(1) $$f\left(x\right)=\frac{a}{\left(1+10^{\left(x-c\right)b}\right)}$$

where, for preferred stimuli, *x* represents the log_10_ concentration, *a* represents the asymptote of the lick scores, *b* represents the slope, and *c* represents the log_10_ concentration one-half *a* (i.e., EC_50_). For nonpreferred stimuli, *a* was set as a constant = 1.0.

For two-bottle test data, the intakes from both 23-h tests for each stimulus were summed. Preference for the stimulus was calculated for each mouse by dividing the intake of the stimulus by total intake (stimulus + water) in the 46-h period. Group means (±SE) for stimulus intake, preference, and total caloric consumption (stimulus + chow) were calculated for each stimulus. These data were compared across groups using mixed two-way ANOVAs.

Latencies for each group in each BFT were used to calculate medians [±SIQR (semi-interquartile range)]. Taste-water licks or taste/water ratios for each stimulus were analyzed in repeated-measures one-way and mixed two-way ANOVAs. Trials initiated for each stimulus were analyzed using *t* tests. For all statistical analyses, a *p* value ≤0.05 was considered statistically significant. Only sessions in which a mouse initiated at least one trial per concentration were included in the concentration-dependent lick analyses for that stimulus, but all sessions were included in analyses of trial number. Further, if a BULBx mouse successfully located the crisp in the postsurgical BFT (criterion, <450 s), it was removed from all analyses. Final group sizes are reported in figure captions.

## Results

**Figure 1.**
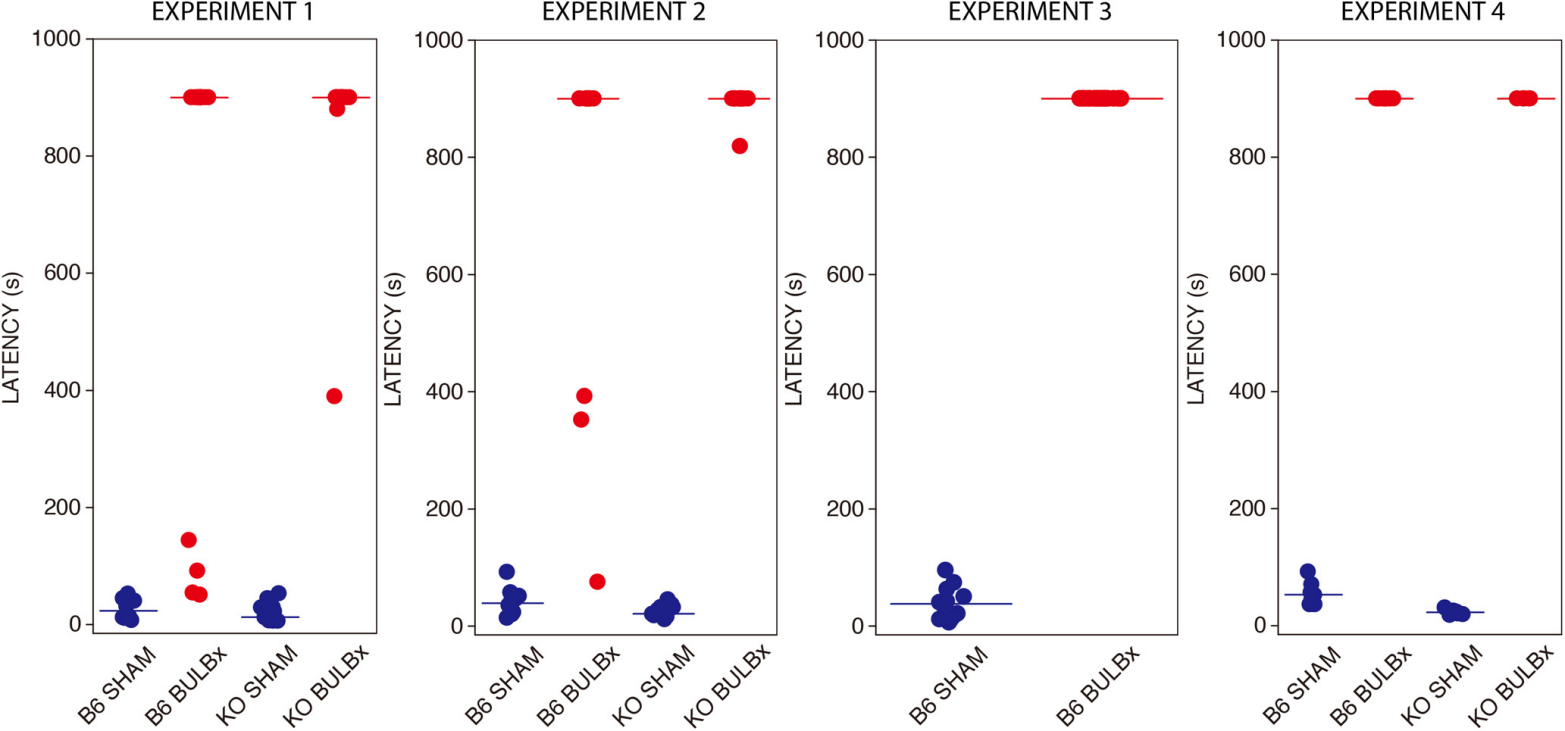
Postsurgical buried food test. Latencies to find the buried potato crisp for each experiment by group (SHAM: blue circles, BULBx: red circles; horizontal lines: median latencies). These tests were performed after the end of behavioral testing. BULBx animals with BFT latencies <450 s were discarded from the behavioral analyses. Mice failing to find the potato crisp during the test underwent a visual test. Median latency (±SIQR) in seconds for the visual test are as follows: experiment 1: B6 BULBx, 9.2 (±9.2); KO BULBx, 11.7 (±8.1); experiment 2: B6 BULBx, 20.4 (±8.7); KO BULBx, 14.5 (±25.8); experiment 3: B6 BULBx, 77.6 (±93.6); experiment 4: B6 BULBx, 81.2 (60.7); KO BULBx, 24.7 (20.4). These mice all passed the presurgical BFT with a latency <110 s.

In all experiments, only BULBx mice that failed the BFT administered after all licking tests were completed (Fig. 1) were included in the behavioral analyses. Importantly, all mice that failed the postsurgical BFT rapidly engaged a crisp during a visual (nonburied) version of the test; this demonstrates that failure to find the buried crisp was not due to a general motivational deficit (Fig. 1).

### Experiment 1: bulbotomy severely blunts concentration-dependent licking of Maltrin and sucrose in brief access tests

Maltodextrins such as Maltrin are discriminable from sucrose by rodents (Nissenbaum and Sclafani, 1987; Smith and Spector, 2017). Both sucrose and maltodextrin are stimuli for which rodents display strong preferences in two-bottle intake tests and avidly lick in a concentration-dependent manner in brief access tests. Although genetic deletion of the T1R2+T1R3 “sweet” taste receptor severely blunts motivated responsiveness to sucrose as assessed in these tests, it has little effect on such behavior when maltodextrin is the stimulus (Sclafani and Clyne, 1987; Sclafani and Mann, 1987; Treesukosol et al., 2009, 2011b; Zukerman et al., 2009b). If the T1R2+T1R3 is necessary for maltodextrin taste but responsiveness in the KO mice can be maintained by smell, then bulbotomy should eliminate concentration-dependent licking to this stimulus in KO mice but not wild-type mice. Accordingly, we tested whether B6 and T1R2+T1R3 KO mice would still display normal licking behavior to these motivationally potent taste stimuli after bulbotomy.

**Figure 2.**
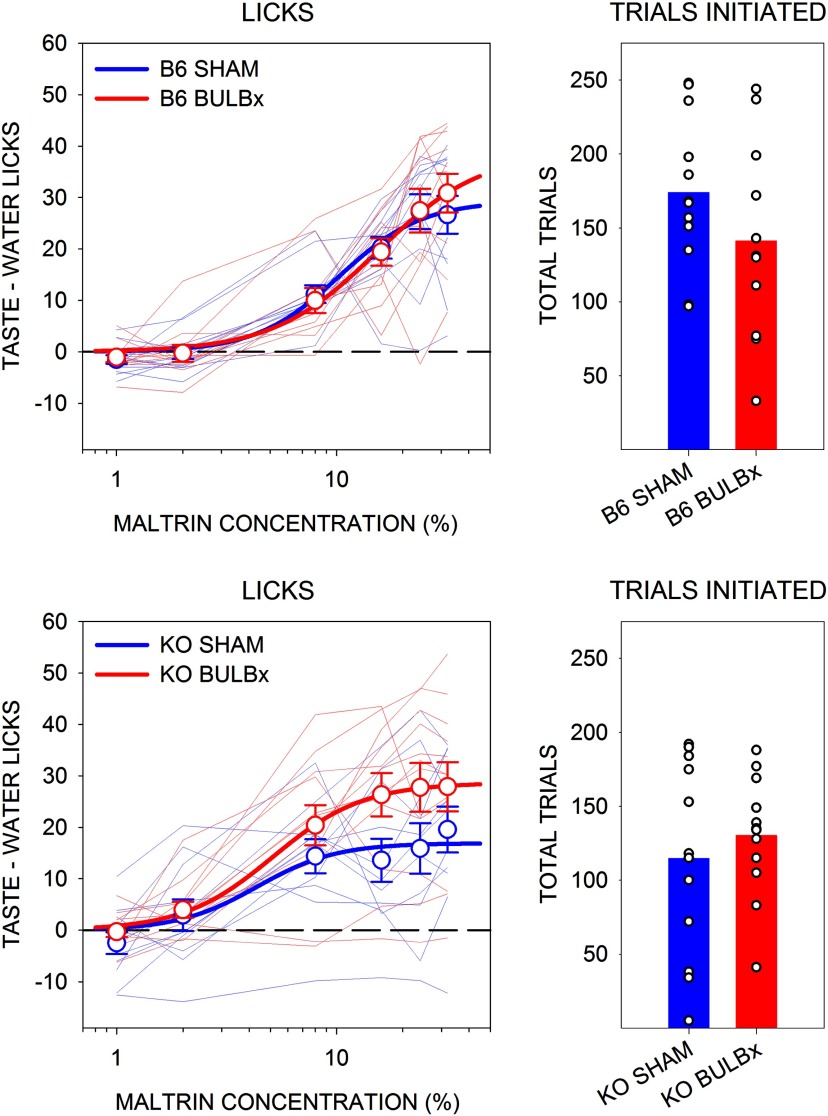
Mean (±SE) presurgical lick scores (left) and the mean total number of taste trials initiated (right) in response to Maltrin by B6 (top) and KO (bottom) mice in experiment 1. SHAM groups are shown in blue, and BULBx groups are shown in red. Lighter blue and red lines represent lick scores of individual mice. Only sessions with at least one trial per concentration were included for lick analyses. B6 SHAM, *n* = 12; B6 BULBx, *n* = 11; KO SHAM, *n* = 11; KO BULBx, *n* = 12. Licks to each concentration are standardized to the mean (±SE) licks to water: B6 SHAM, 13.2 ± 1.0; B6 BULBx, 12.7 ± 1.2; KO SHAM, 22.1 ± 3.4; KO BULBx, 18.1 ± 1.8. See Tables 1 and 2 for outcomes of statistical tests.

**Figure 3.**
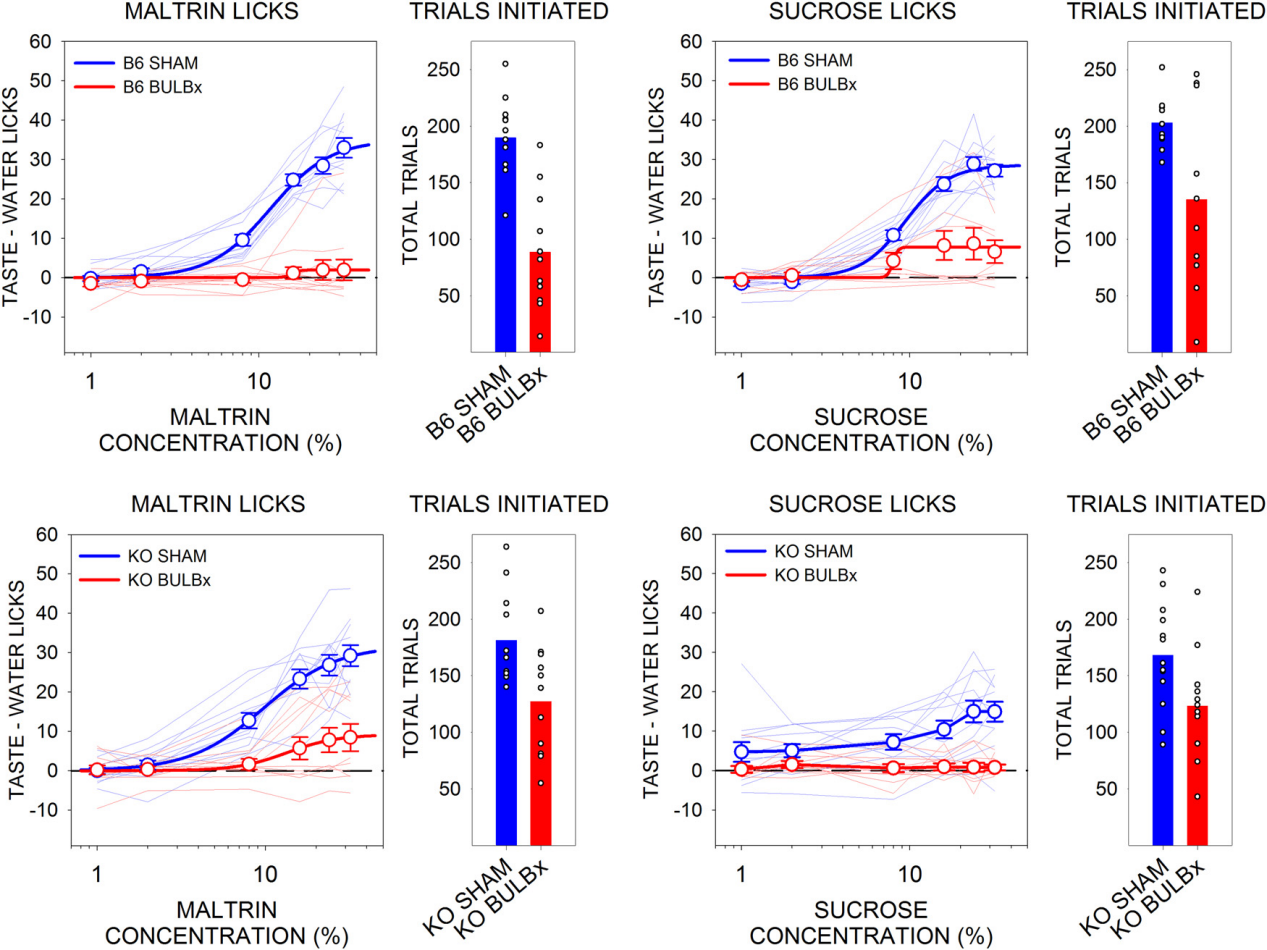
Mean (±SE) postsurgical lick scores and the mean (±SE) total number of taste trials initiated to Maltrin and sucrose by B6 (top) and KO (bottom) mice in experiment 1. SHAM groups are shown in blue, and BULBx groups are shown in red. Lighter blue and red lines represent lick scores of individual mice. Only sessions with at least one trial per concentration were included for lick analyses. Group sizes for Maltrin were as follows: B6 SHAM, *n* = 12; B6 BULBx, *n* = 11; KO SHAM, *n* = 12; KO BULBx, *n* = 12. Group sizes for sucrose were as follows: B6 SHAM, *n* = 12; B6 BULBx, *n* = 10; KO SHAM, *n* = 12; KO BULBx, *n* = 12. Licks to each concentration are standardized to the mean (±SE) number of licks to water. Maltrin water licks were as follows: B6 SHAM, 12.1 ± 0.9; B6 BULBx, 11.5 ± 2.4; KO SHAM, 17.7 ± 1.9; KO BULBx, 15.9 ± 2.6. Sucrose water licks were as follows: B6 SHAM, 12.1 ± 1.1; B6 BULBx, 12.5 ± 2.4; KO SHAM, 24.3 ± 3.1; KO BULBx, 13.4 ± 2.4. See Tables 1 and 2 for outcomes of statistical tests.

Presurgically, there were no differences in the concentration–response functions for Maltrin licking between the respective surgical groups (Table 1, Fig. 2) or any differences between the number of trials initiated (Table 1, Fig. 2). In striking contrast, bulbotomy severely suppressed concentration-dependent licking to Maltrin in both B6 and KO mice and significantly decreased the number of trials initiated (Tables 1, 2, Fig. 3).

**Table 1:** Experiment 1 comparisons of lick data between surgical groups

	Surgery	Concentration	Surgery × Concentration
B6			
Maltrin			
Presurgery	*F*_(1,21)_ = 0.05; *p* = 0.82	*F*_(5,105)_ = 61.9; *p* < 0.01	*F*_(5,105)_ = 0.34; *p* = 0.89
Postsurgery	*F*_(1,21)_ = 117.09; *p* < 0.01	*F*_(5,105)_ = 62.72; *p* < 0.01	*F*_(5,105)_ = 41.23; *p* < 0.01
Sucrose	*F*_(1,20)_ = 22.7; *p* < 0.01	*F*_(5,100)_ = 69.1; *p* < 0.01	*F*_(5,100)_ = 22.8; *p* < 0.01
KO			
Maltrin			
Presurgery	*F*_(1,22)_ = 3.09; *p* = 0.09	*F*_(5,110)_ = 30.5; *p* < 0.01	*F*_(5,110)_ = 1.64; *p* = 0.16
Postsurgery	*F*_(1,22)_ = 24.21; *p* < 0.01	*F*_(5,110)_ = 52.69; *p* < 0.01	*F*_(5,110)_ = 16.44; *p* < 0.01
Sucrose	*F*_(1,22)_ = 21.4; *p* < 0.01	*F*_(5,110)_ = 6.9; *p* < 0.01	*F*_(5,110)_ = 7.0; *p* < 0.01

**Table 2:** Two-sample *t* tests comparing trials initiated by SHAM vs BULBx mice by phase and experiment

	B6 vs BULBx	KO vs BULBx
Experiment 1		
Maltrin		
Presurgery	*t*_(21)_ = 1.31; *p* = 0.21	*t*_(22)_ = 0.69; *p* = 0.49
Postsurgery	*t*_(21)_ = 5.43; *p* < 0.01	*t*_(22)_ = 3.07; *p* < 0.01
Sucrose	*t*_(21)_ = 2.753; *p* = 0.02	*t*_(22)_ = 2.30; *p* = 0.03
Experiment 2		
Quinine	*t*_(22)_ = 2.49; *p* = 0.03	*t*_(23)_ = 1.01; *p* = 0.32
Citric acid	*t*_(22)_ = 1.40; *p* = 0.16	*t*_(23)_ = 2.47; *p* = 0.03
Experiment 3		
Intralipid		
Presurgery	*t*_(29)_ = 0.04; *p* = 0.97	
Postsurgery	*t*_(29)_ = 4.35; *p* < 0.01	
Sucrose	*t*_(29)_ = 0.49; *p* = 0.631	
Saccharin	*t*_(29)_ = 0.71; *p* = 0.49	

The severely impaired responsiveness to Maltrin in BULBx mice across the entire range of concentrations was also observed when sucrose was the stimulus (Table 1, Fig. 3). As was the case with Maltrin, there was a reduction in the number of trials initiated on the sucrose test in BULBx mice (Table 2, Fig. 3). As expected, there was also a genotype effect on sucrose licking, with SHAM KO mice licking significantly less than SHAM B6 mice (genotype: *F*_(1,22)_ = 6.63, *p* = 0.012; concentration: *F*_(5,110)_ = 88.54; *p* < 0.01; genotype × concentration: *F*_(5,110)_ = 23.56, *p* < 0.01). However, with the large reduction in licking following bulbotomy, there was no main effect of genotype between the BULBx groups (genotype: *F*_(1,20)_ = 3.57, *p* = 0.08; concentration: *F*_(5,100)_ = 4.17; *p* < 0.01; genotype × concentration: *F*_(5,100)_ = 4.10, *p* < 0.01). Moreover, BULBx KO mice did not display any concentration-dependent increases in sucrose licking (*F*_(5,55)_ = 0.273, *p* = 0.93), but SHAM KO mice did (*F*_(5,45)_ = 4.13, *p* < 0.01), albeit severely attenuated relative to SHAM B6 mice.

### Experiment 2: bulbotomy has no effect on concentration-dependent lick avoidance of quinine and citric acid in brief access tests

**Figure 4.**
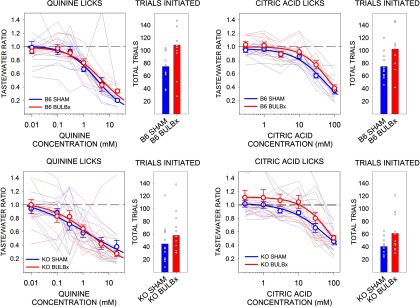
Mean (±SE) postsurgical taste/water ratios and the mean (±SE) total number of taste trials initiated to quinine and citric acid by B6 (top) and KO (bottom) mice in experiment 2. SHAM groups are shown in blue, and BULBx groups are shown in red. Lighter blue and red lines represent taste/water ratios of individual mice. Only sessions with at least one trial per concentration were included for lick analyses. Group sizes for quinine were as follows: B6 SHAM, *n* = 12; B6 BULBx, *n* = 9; KO SHAM, *n* = 13; KO BULBx, *n* = 11. Group sizes for citric acid were as follows: B6 SHAM, *n* = 12; B6 BULBx, *n* = 8; KO SHAM, *n* = 13; KO BULBx, *n* = 12. Licks to each concentration are standardized to the mean (±SE) licks to water. Quinine water licks were as follows: B6 SHAM, 53.8 ± 6.8; B6 BULBx, 49.5 ± 2.1; KO SHAM, 63.22 ± 3.4; KO BULBx, 60.3 ± 4.3. Citric acid water licks were as follows: B6 SHAM, 59.8 ± 2.5; B6 BULBx, 50.7 ± 1.3; KO SHAM, 67.87 ± 2.8; KO BULBx, 57.9 ± 5.5. See Tables 2 and 3 for outcomes of statistical tests.

Because, after bulbotomy, KO and B6 mice were severely impaired in their concentration-dependent responsiveness to both Maltrin and sucrose, two highly preferred stimuli with positive hedonic valence, we tested the responsiveness of BULBx and SHAM KO and B6 mice to two normally avoided stimuli with negative hedonic valence, quinine and citric acid. There was no effect of bulbotomy on the concentration-dependent licking to either quinine (Table 3, Fig. 4) or citric acid (Table 3, Fig. 4) in either B6 or KO mice. BULBx B6 mice initiated more quinine trials than SHAM B6 mice, and BULBx KO mice initiated more citric acid trials than SHAM KO mice. Notwithstanding these differences, all groups initiated many trials (Table 4, Fig. 4).

**Table 3:** Experiment 2 comparisons of lick data between surgical groups

	Surgery	Concentration	Surgery × Concentration
B6			
Quinine	*F*_(1,19)_ = 0.20; *p* = 0.66	*F*_(5,95)_ = 76.28; *p* < 0.01	*F*_(5,95)_ = 1.19; *p* = 0.32
Citric acid	*F*_(1,18)_ = 1.8; *p* = 0.20	*F*_(5,90)_ = 112.38; *p* < 0.01	*F*_(5,90)_ = 1.36; *p* = 0.25
KO			
Quinine	*F*_(1,22)_ = 0.03; *p* = 0.87	*F*_(5,110)_ = 34.11; *p* < 0.01	*F*_(5,110)_ = 0.88; *p* = 0.50
Citric acid	*F*_(1,23)_ = 1.85; *p* = 0.19	*F*_(5,115)_ = 57.29; *p* < 0.01	*F*_(5,115)_ = 0.48; *p* = 0.79

**Table 4:** Experiment 3 comparisons of lick data between surgical groups

	Surgery	Concentration	Surgery × Concentration
Intralipid			
Presurgery	*F*_(1,28)_ = 0.04; *p* = 0.85	*F*_(5,140)_ = 49.21; *p* < 0.01	*F*_(5,140)_ = 0.17; *p* = 0.97
Postsurgery	*F*_(1,20)_ = 32.24; *p* < 0.01	*F*_(5,100)_ = 23.04; *p* < 0.01	*F*_(5,100)_ = 7.35; *p* < 0.01
Sucrose	*F*_(1,26)_ = 24.38; *p* < 0.01	*F*_(5,130)_ = 102.51; *p* < 0.01	*F*_(5,130)_ = 6.87; *p* < 0.01
Saccharin	*F*_(1,27)_ = 15.20; *p* < 0.01	*F*_(5,135)_ = 72.43; *p* < 0.01	*F*_(5,135)_ = 2.88; *p* = 0.02

### Experiment 3: bulbotomy blunts concentration-dependent licking of Intralipid and sodium saccharin in brief access tests

In this experiment, we tested the generality of the effect of bulbotomy on preferred oral stimuli that have positive hedonic valence in B6 mice. We chose Intralipid because it is a noncarbohydrate stimulus that is highly preferred by rodents and has calories. In addition, we tested sodium saccharin, a noncaloric sweetener, and sucrose; the latter was included to confirm the initial observations in experiment 1.

**Figure 5.**
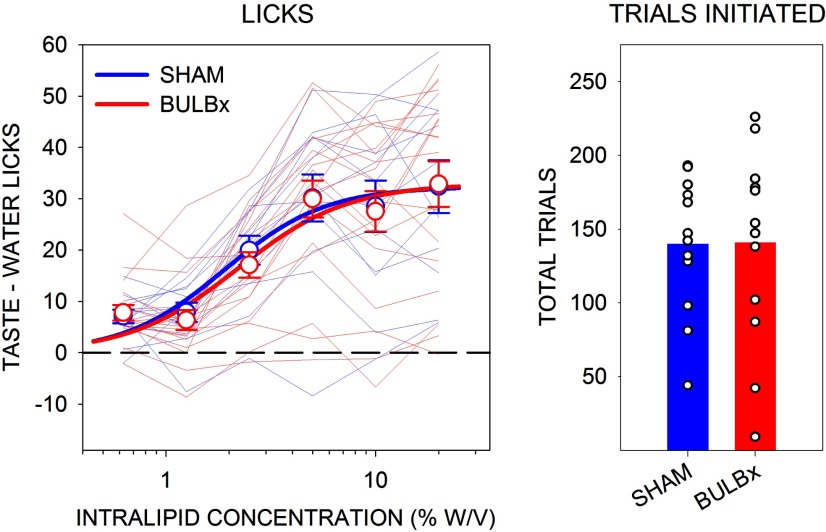
Mean (±SE) presurgical Lick Scores (left) and mean total number of taste trials initiated (right) to Intralipid by B6 mice in experiment 3. SHAM groups are shown in blue, and BULBx groups are shown in red. Lighter blue and red lines represent lick scores of individual mice. Only sessions with at least one trial per concentration were included for lick analyses. SHAM, *n* = 12; BULBx, *n* = 18. Licks to each concentration are standardized to the mean (±SE) licks to water: SHAM, 15.2 ± 0.8; BULBx, 14.5 ± 1.2. See Tables 2 and 4 for outcomes of statistical tests.

**Figure 6.**
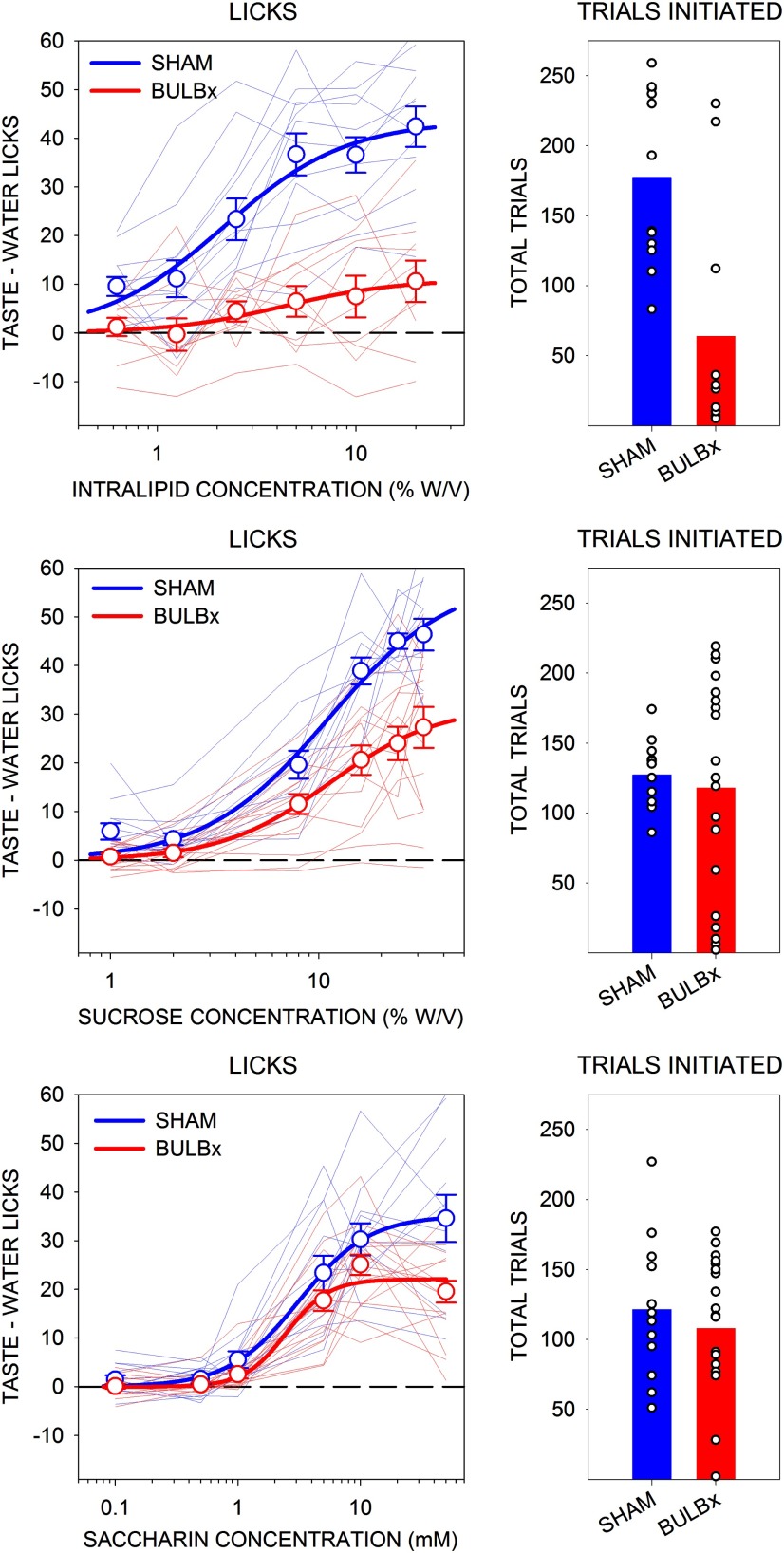
Mean (±SE) postsurgical lick scores (left) and the mean total number of taste trials initiated (right) to Intralipid (top), sucrose (middle), and saccharin (bottom) by B6 mice in experiment 3. SHAM groups are shown in blue, and BULBx groups are shown in red. Lighter blue and red lines represent lick scores of individual mice. Only sessions with at least one trial per concentration were included for lick analyses, as follows: Intralipid: SHAM, *n* = 12; BULBx, *n* = 11; sucrose: SHAM, *n* = 12; BULBx, *n* = 16; saccharin: SHAM, *n* = 12; BULBx, *n* = 17. Licks to each concentration are standardized to the mean (±SE) licks to water, as follows: Intralipid: SHAM, 12.3 ± 1.0; BULBx, 30.8 ± 4.7; sucrose: SHAM, 8.2 ± 0.9; BULBx, 11.7 ± 1.4; saccharin: SHAM, 8.7 ± 1.4; BULBx, 7.3 ± 0.8. See Tables 2 and 4 for outcomes of statistical tests.

Presurgically, there were no differences in the concentration–response functions for Intralipid licking among the groups (Table 4, Fig. 5) or any differences among the number of trials initiated (Table 1, Fig. 5). However, emulating what was seen with Maltrin in experiment 1, bulbotomy severely suppressed concentration-dependent licking in response to Intralipid in B6 mice (Table 4, Fig. 6). Similar results were found with sucrose, replicating the effect seen in experiment 1 (Table 4, Fig. 6). Bulbotomy also significantly blunted licking in response to Na-saccharin, but this appeared to be less profound than what was seen with Maltrin, sucrose, and Intralipid (Table 4, Fig. 6).

The BULBx mice initiated fewer Intralipid trials postsurgically than SHAM mice (Table 2, Fig. 6). However, there were no differences in the number of trials taken by BULBx and SHAM mice for sucrose and Na-saccharin testing (Table 2, Fig. 6). Thus, overall, although the BULBx mice displayed blunted concentration-dependent licking for the various normally preferred solutions, the fact that they engaged in trial-taking on these tests indicates they were not in a general motivational stupor.

### Experiment 4: bulbotomy does not compromise preference for or intake of high concentrations of sucrose or Maltrin in 23 h two-bottle tests

**Figure 7.**
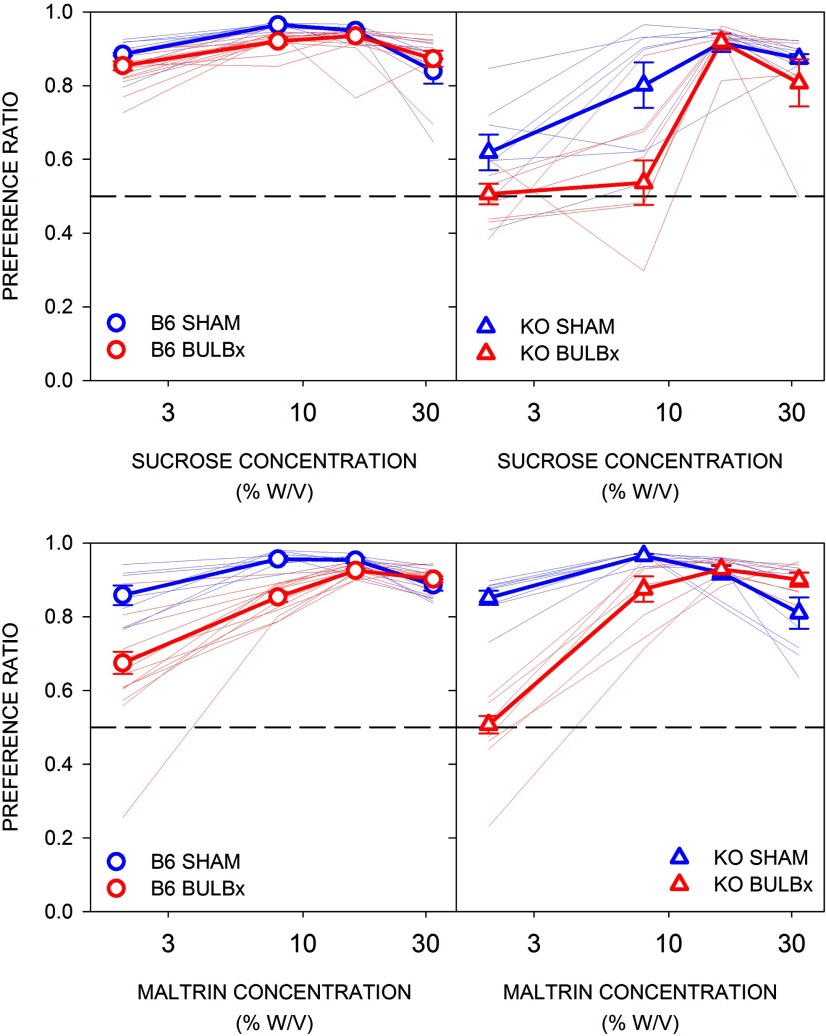
Mean (±SE) preference ratios to sucrose (top) and Maltrin (bottom) by B6 (left, circles) and KO (right, triangles) mice in experiment 4. SHAM groups are shown in blue, and BULBx groups are shown in red. Lighter blue and red lines represent preference ratios of individual mice. Preference ratios are calculated by dividing intake of the solution by total intake (solution + water). B6 SHAM, *n* = 8; B6 BULBx, *n* = 13; KO SHAM, *n* = 8; KO BULBx, *n* = 7. See Tables 5 and 6 for outcomes of statistical tests.

**Figure 8.**
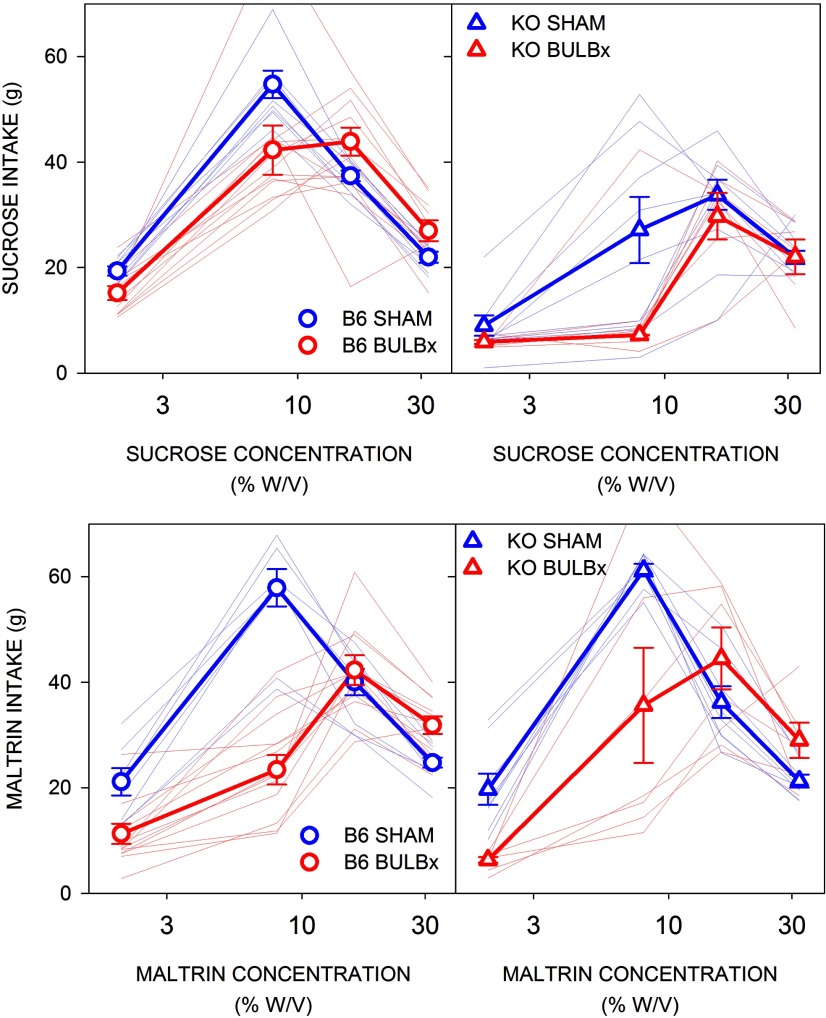
Mean (±SE) intake of sucrose (top) and Maltrin (bottom) by B6 (left, circles) and KO (right, triangles) mice in experiment 4. SHAM groups are shown in blue, and BULBx groups are shown in red. Lighter blue and red lines represent intakes of individual mice. B6 SHAM, *n* = 8; B6 BULBx, *n* = 13; KO SHAM, *n* = 8; KO BULBx, *n* = 7. See Tables 5 and 6 for outcomes of statistical tests.

A separate cohort of B6 and KO mice was tested in a series of 23-h two-bottle preference tests (two tests per concentration) with four concentrations, spanning low to high, of Maltrin and sucrose (chemical order counterbalanced). Both the preference for and the intake of Maltrin by both B6 and KO mice were significantly lower in BULBx mice relative to SHAM mice at the two lower concentrations (Tables 5, 6, Figs. 7, 8). However, at the two higher concentrations, there were no differences between the surgical groups, within either genotype, in preference or intake except that B6 BULBx mice actually drank significantly more Maltrin than SHAM mice.

**Table 5:** Experiment 4 comparisons of preference and intake between surgical groups

	Surgery	Concentration	Surgery × Concentration
B6 preference			
Sucrose	*F*_(1,19)_ = 4.1; *p* = 0.06	*F*_(3,57)_ = 17.2; *p* < 0.01	*F*_(3,57)_ = 2.1; *p* = 0.12
Maltrin	*F*_(1,19)_ = 18.3; *p* < 0.01	*F*_(3,57)_ = 34.7; *p* < 0.01	*F*_(3,57)_ = 12.5; *p* < 0.01
KO preference			
Sucrose	*F*_(1,13)_ = 7.3; *p* = 0.02	*F*_(3,39)_ = 29.5; *p* < 0.01	*F*_(3,39)_ = 2.5; *p* = 0.07
Maltrin	*F*_(1,13)_ = 16.5; *p* < 0.01	*F*_(3,39)_ = 48.1; *p* < 0.01	*F*_(3,39)_ = 34.4; *p* < 0.01
B6 intake			
Sucrose	*F*_(1,19)_ = 0.5; *p* = 0.47	*F*_(3,57)_ = 88.1; *p* < 0.01	*F*_(3,57)_ = 7.1; *p* < 0.01
Maltrin	*F*_(1,19)_ = 10.9; *p* < 0.01	*F*_(3,57)_ = 75.3; *p* < 0.01	*F*_(3,57)_ = 38.9; *p* < 0.01
KO intake			
Sucrose	*F*_(1,13)_ = 2.8; *p* = 0.11	*F*_(3,39)_ = 19.5; *p* < 0.01	*F*_(3,39)_ = 2.1; *p* = 0.12
Maltrin	*F*_(1,13)_ = 4.19; *p* = 0.06	*F*_(3,39)_ = 33.0; *p* < 0.01	*F*_(3,39)_ = 9.7; *p* < 0.01

**Table 6:** Experiment 4 comparisons of preference and intake between surgical groups at each concentration for each stimulus

Stimulus	Preference	Intake
B6 vs BULBx		
2% sucrose	*t*_(19)_ = 2.22; *p* = 0.04	*t*_(19)_ = 2.88; *p* = 0.01
8% sucrose	*t*_(19)_ = 3.09; *p* < 0.01	*t*_(19)_ = 2.31; *p* = 0.03
16% sucrose	*t*_(19)_ = 1.23; *p* = 0.23	*t*_(19)_ = 1.18; *p* = 0.25
32% sucrose	*t*_(19)_ = 0.70; *p* = 0.50	*t*_(19)_ = 2.29; *p* = 0.03
2% Maltrin	*t*_(19)_ = 3.68; *p* < 0.01	*t*_(19)_ = 3.39; *p* < 0.01
8% Maltrin	*t*_(19)_ = 6.15; *p* < 0.01	*t*_(19)_ = 6.98; *p* < 0.01
16% Maltrin	*t*_(19)_ = 1.34; *p* = 0.13	*t*_(19)_ = 0.73; *p* = 0.48
32% Maltrin	*t*_(19)_ = 1.44; *p* = 0.17	*t*_(19)_ = 3.22; *p* < 0.01
KO SHAM vs BULBx		
2% sucrose	*t*_(13)_ = 2.22; *p* = 0.04	*t*_(13)_ = 1.54; *p* = 0.14
8% sucrose	*t*_(13)_ = 2.30; *p* = 0.04	*t*_(13)_ = 1.81; *p* = 0.09
16% sucrose	*t*_(13)_ = 0.12; *p* = 0.91	*t*_(13)_ = 0.72; *p* = 0.48
32% sucrose	*t*_(13)_ = 0.95; *p* = 0.36	*t*_(13)_ = 0.10; *p* = 0.92
2% Maltrin	*t*_(13)_ = 8.32; *p* < 0.01	*t*_(13)_ = 4.73; *p* < 0.01
8% Maltrin	*t*_(13)_ = 3.25; *p* < 0.01	*t*_(13)_ = 3.13; *p* < 0.01
16% Maltrin	*t*_(13)_ = 0.36; *p* = 0.73	*t*_(13)_ = 0.98; *p* = 0.35
32% Maltrin	*t*_(13)_ = 1.79; *p* = 0.10	*t*_(13)_ = 1.92; *p* = 0.08

For sucrose, B6 BULBx mice showed slightly reduced preference for and lower intake of the two lowest concentrations (Tables 5, 6, Figs. 7, 8). In fact, like what was seen with Maltrin, the BULBx B6 mice actually drank more sucrose compared with the SHAM group at the highest concentration. BULBx KO mice had significantly lower sucrose preference at the two lower concentrations compared with their SHAM counterparts, but intake did not significantly differ between the surgical groups. At the two higher concentrations, there were no differences in sucrose intake or preference between the BULBx and SHAM KO mice.

**Figure 9.**
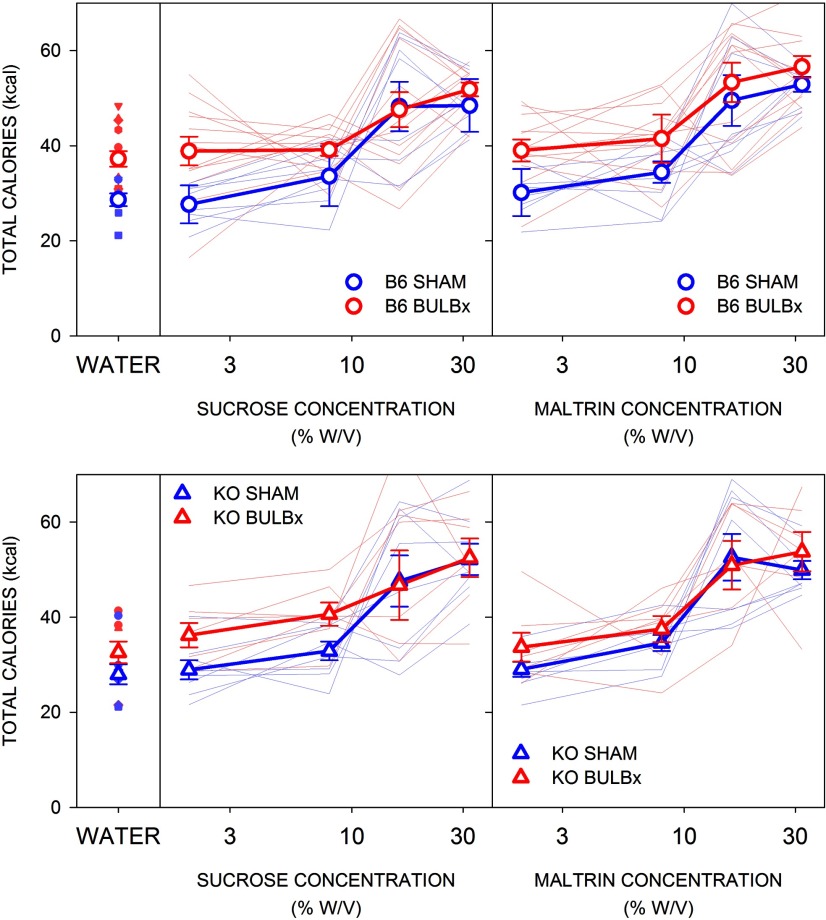
Mean (±SE) total caloric intake during two-bottle testing with water (left), Maltrin (middle), and sucrose (right) by B6 (left, circles) and KO (right, triangles) mice in experiment 4. SHAM groups are shown in blue, and BULBx groups are shown in red. Lighter blue and red lines represent total caloric intakes of individual mice. B6 SHAM, *n* = 8; B6 BULBx, *n* = 13; KO SHAM, *n* = 8; KO BULBx, *n* = 7. See Table 7 for outcomes of statistical tests.

Across all the preference tests (Maltrin and sucrose), B6 BULBx mice consumed more total daily calories (including chow intake) and KO BULBx mice consumed a similar number of total daily calories as their SHAM counterparts, regardless of sugar, and all mice increased caloric intake as a function of concentration (Table 7, Fig. 9). The same was found during the initial two water-only days (B6: *t*_(19)_ = 3.32, *p* < 0.01; KO: *t*_(13)_ = 1.62, *p* = 0.13; Fig. 9).

**Table 7:** Experiment 4 comparisons of caloric intake between surgical groups

	Surgery	Concentration	Surgery × Concentration
B6			
Sucrose	*F*_(1,19)_ = 4.3; *p* = 0.05	*F*_(3,57)_ = 18.9; *p* < 0.01	*F*_(3,57)_ = 1.7; *p* = 0.17
Maltrin	*F*_(1,19)_ = 5.5; *p* = 0.03	*F*_(3,57)_ = 25.9; *p* < 0.01	*F*_(3,57)_ = 1.0; *p* = 0.38
KO			
Sucrose	*F*_(1,13)_ = 2.1; *p* = 0.18	*F*_(3,39)_ = 16.6; *p* < 0.01	*F*_(3,39)_ = 0.5; *p* = 0.68
Maltrin	*F*_(1,13)_ = 1.5; *p* = 0.24	*F*_(3,39)_ = 23.7; *p* < 0.01	*F*_(3,39)_ = 0.2; *p* = 0.92

These results reveal that BULBx mice are not generally unmotivated. Indeed, they are quite capable of approaching and avidly ingesting sucrose and Maltrin at higher concentrations in long-term tests when they can take advantage of any orosensory cues available and even bottle position to guide their behavior over time. At lower concentrations, these cues are less salient, and thus the consequences of bulbotomy on the behavior are more apparent. They are most apparent when the animals are given the brief access test in which the immediate responses to small volumes of stimuli are measured. Under those conditions, the potency of the stimuli to drive licking behavior is severely compromised by the surgical isolation of the olfactory bulbs. This effect, however, is limited to stimuli of positive affective valence, because BULBx mice display normal taste-guided licking avoidance of citric acid and quinine.

### Histology

**Figure 10.**
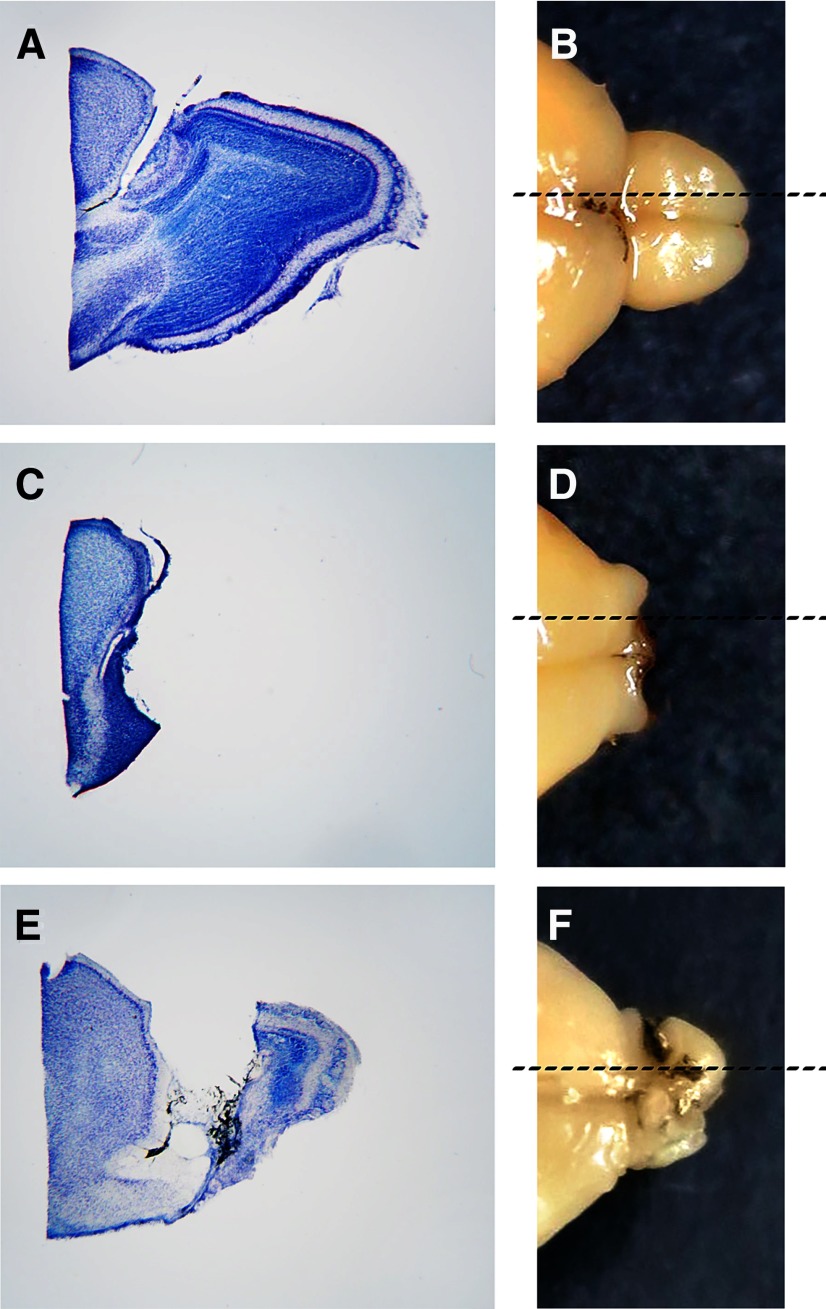
***A–F***, Representative photomicrographs of a sagittal brain section through the olfactory bulb and rostral pole of the forebrain stained with thionin (***A***, ***C***, ***E***) and photographs of the accompanying whole brain from which the section was derived (***B***, ***D***, ***F***). Dashed lines in ***B***, ***D***, and ***F*** represent the approximate medial–lateral planes of the Nissl-stained sections in ***A***, ***C***, and ***E***. ***A***, ***B***, intact olfactory bulbs from a SHAM mouse. ***C***, ***D***, complete separation of the olfactory bulbs from the rostral forebrain (olfactory bulbs not shown) in a BULBx mouse. ***E***, ***F***, incomplete separation of the olfactory bulb from the rostral forebrain in a BULBx mouse that passed the BFT and was discarded from the analysis.

All BULBx mice in all four experiments that had failed the BFT had histologically confirmed transections. Figure 10*C* shows the photomicrograph of a representative sagittal section of the rostral pole of a brain (Mouse GQ03, experiment 1) in which the olfactory bulb was completely transected (Fig. 10, compare ***A***, ***E***, ***C***). Figure 10, *C* and *E*, displays a representative sagittal section of brain from a mouse receiving bulbotomy surgery that failed (Mouse GQ03, experiment 1) and a mouse that passed the BFT (Mouse GQ17, experiment 1), respectively. We did the best we could to prevent inadvertent separation of the olfactory tract during histologic processing, but we cannot entirely rule out that it occurred. For this reason, we based our BULBx inclusion criteria on the BFT outcomes.

## Discussion

It is clear from these results that the unconditioned motivational salience of oral stimuli that have a positive affective valence is largely dependent on intact anatomic connections between the olfactory bulb and its associated central sites. The concentration-dependent licking of the maltodextrin Maltrin, the disaccharide sucrose, and the fat emulsion Intralipid was substantially suppressed by bulbotomy. While the blunting of concentration-dependent licking of sucrose and saccharin in experiment 3 was apparently not as severe as that seen for sucrose in experiment 1, it is nonetheless evident both graphically and statistically. This may be due to differences in the explicit testing history of the animals between the two experiments; responsiveness in the brief access test can be influenced by prior testing experience (Treesukosol et al., 2009, 2011a). Importantly, and in contrast to what was seen with normally preferred taste stimuli, the unconditionally negative affective valence of avoided compounds appeared to be relatively impervious to this anatomic disconnection of the olfactory and vomeronasal systems. The concentration-dependent decrease in licking of quinine and citric acid was not significantly affected by bulbotomy. Likewise, the bulbotomy left the preference and intake of the higher concentrations of both Maltrin and sucrose unperturbed as assessed by two-bottle long-term tests. Because the downstream connections of both the main and accessory olfactory bulb were transected, we cannot distinguish whether one structure is more critical than the other with respect to the effects of the surgery on appetitive and consummatory responses to the preferred stimuli observed here. Nevertheless, the dissociation of the functional consequences of this neural insult on the main and accessory olfactory bulbs along the dimensions of positive and negative taste-evoked affect is compelling.

The double KO mice lacking both subunits of the T1R2+T1R3 heterodimeric taste receptor that had sham surgery responded as expected. They displayed relatively normal responsiveness to maltodextrin, but exhibited severely attenuated licking of sucrose, which was, interestingly, further suppressed by bulbotomy. It is not unusual for T1R KO animals to display some degree of concentration-dependent responsiveness to sugars in the brief access test when they have had sufficient prior testing experience with nutritive stimuli (Treesukosol et al., 2009, 2011a,b). This is thought to be due to the animals forming an association with when remaining detectable orosensory cues are present in the stimulus (e.g., viscosity, smell) and the positive nutritive effects from ingestion across entire sessions of trials. The fact that bulbotomy eliminated this may lead one to postulate that the KO animals were using an olfactory cue to form this association, but the observation that sucrose licking by bulbotomized B6 mice, which have an intact T1R2+T1R3 heterodimer, was also severely curbed relative to their sham-operated counterparts, supports other possibilities (more on this below). Likewise, the fact that bulbotomy substantially curtailed licking to Maltrin in KO and B6 mice does not necessarily mean that explicit gustatory signals evoked by this maltodextrin do not exist or are not important in driving responsiveness (Zukerman et al., 2013; Sclafani and Ackroff, 2014).

Others have found that disruptive manipulations of the olfactory system in rodents can decrease preferences for taste stimuli in long-term tests, but such effects have not been systematically studied, are often limited to low concentrations, and are not universally observed (Vigorito and Sclafani, 1987; Ramirez, 1993; Stock et al., 2000; Takeda et al., 2001; Uebayashi et al., 2001; Chambliss et al., 2004; Slattery et al., 2007; Romeas et al., 2009; Zukerman et al., 2009a; Sato et al., 2010). Here, we found decreases in the preference for and intake of Maltrin and, to some extent, sucrose (KO mice were more affected than B6 mice) in BULBx mice at low concentrations of these carbohydrate stimuli in 23-h two-bottle tests. However, at high concentrations, these mice displayed quite avid ingestion of and normal preference for these solutions. Although long-term two-bottle intake tests are commonly used as a first approximation of the behavioral responsiveness of an animal to a given chemical stimulus, they are not considered a rigorous assessment of taste function because choice and amount consumed can be significantly influenced by nongustatory factors, especially those of a postoral nature (Spector, 2003). Indeed, we believe that the remaining orosensory input in tandem with postingestive signals as well as potential bottle position cues influenced the ingestive behavior of the BULBx animals over the 23-h periods. The brief access test largely circumvents the interpretive limitations of long-term intake tests and more purely focuses on the orosensory affective characteristics of the stimulus; it is not unusual for manipulations of the gustatory system to produce different outcomes between the two testing paradigms. That said, Uebayashi et al. (2001) used a brief access test to compare the responsiveness of water-restricted mice to an array of basic taste stimuli before and after the induction of anosmia via irrigation of the nasal epithelium with zinc sulfate. In contrast to what we found, after nasal zinc sulfate treatment, mice did not alter their lick rates to sucrose (or NaCl), but no longer displayed licking avoidance of quinine and HCl. The failure of the treatment to affect sucrose licking could possibly be due to the fact that the water-restricted mice were likely licking both water and sucrose at near maximal rates. However, with respect to responsiveness to unconditionally aversive tastes, the disparity between our results and those of Uebayashi et al. (2001) is harder to explain, especially given that bulbotomy is an even more severe offense to the olfactory system compared with nasal zinc sulfate. Nevertheless, it is quite clear that concentration-dependent avoidance of the prototypical bitter ligand quinine and the prototypical acid stimulus citric acid (sour to humans) did not statistically differ between bulbotomy sham-operated mice regardless of genotype.

Bulbectomy, a procedure in which the olfactory bulbs are entirely ablated, in rodents is considered by some to be an animal model of clinical depression (Willner, 1990; Kelly et al., 1997; Stock et al., 2000; Wrynn et al., 2000; Cryan and Mombereau, 2004; Song and Leonard, 2005; Yurttas et al., 2017; Ruda-Kucerova et al., 2018). This view arises from the constellation of behavioral and neurochemical consequences of the manipulation, some of which are reversed by treatment with antidepressant drugs. Although we did not remove the olfactory bulbs, our bulbotomy procedure would possibly lead to similar outcomes if tested. We do not claim that bulbotomy does not have an effect on a diverse set of behaviors. Naturally, such a major loss of sensory systems on which rodents heavily rely along with removal of such a large body of central projections, in addition to depriving neurons in central nuclei providing centrifugal fibers innervating the bulb of their target, would be expected to affect a variety of behaviors and neurotransmitter systems. However, our interest here lies in whether taste-guided behavior is actually affected by bulbotomy, given the limited and uneven literature on this specific aspect of sensory function. Moving forward, given the outcomes presented here, it would be logical to explore the way that chemosensory circuits are affected by the anatomic disconnection of the olfactory bulbs from the rest of the brain rendering normally rewarding taste stimuli motivationally impotent; this is not explicitly apparent but is important to understand.

Over and beyond questions about its validity (Cryan and Mombereau, 2004), the bulbectomy model of depression in mice does not serve as an explanation in and of itself for the behavioral effects observed here. For example, the behavior of the B6 BULBx mice to sucrose remarkably resembled that of the KO SHAM mice in the tests used here, but it would be difficult to support any claim that the genetic deletion of the T1R2+T1R3 heterodimer is a model of clinical depression. Moreover, based on the constellation of results reported here, it does not appear that bulbotomized mice are simply and generally unmotivated. When driven by a water-deprived state, BULBx mice initiated just as many, or, in some cases significantly more, trials in the brief access test. The basis of this increase in trials remains to be understood, but it certainly demonstrates that under these particular test conditions, BULBx mice are willing to approach the drinking spout. When given ample opportunity to repeatedly sample and ingest sucrose or Maltrin in the home cage versus water, BULBx mice drank amounts of the high concentrations comparable to SHAM mice and showed preferences for the higher concentrations of these solutions that were similar to that seen in the surgical controls. Indeed, the total caloric intake of B6 BULBx mice significantly surpassed that seen in their SHAM counterparts and did not differ between the surgical KO groups over the course of two-bottle testing. Thus, an overall decrease to express motivated approach and ingestive behavior was not observed after bulbotomy. Rather, it was when the animals were confronted with very short trials and responses were based on relatively small stimulus samples that the severe consequences of the neural isolation of the olfactory bulbs on concentration-dependent licking of positively valenced taste solutions were fully revealed.

While the behavioral effects of bulbotomy were clear, they were not without variability among subjects. Of course, individual differences on behavioral measures after a given neural manipulation are not uncommon. This is because behavior is the final readout of nervous system function allowing for the accumulated influence of variation in any number of relevant processes leading to the outcome. The variability observed within functionally and histologically confirmed bulbotomized groups indicates that this surgery does not necessarily render these animals entirely unresponsive to taste compounds with positive affective valence. Obviously, there are several factors that determine the nature of licking behavior during a brief access test; brain connections with the olfactory bulbs simply represent one contribution, but clearly a very important one.

One way to view the mechanisms underlying the behavioral consequences of bulbotomy on taste responsiveness is from a psychophysical vantage point. The brief access test relies on the affective characteristics of the taste stimulus to drive licking behavior or avoidance and does not plainly reveal the sensory-discriminative properties of the stimulus in terms of perceived quality or intensity (St John and Spector, 2008). We cannot rule out that the bulbotomy-induced decreases in affective responsiveness to the preferred taste compounds in our study were secondary to a significant decrease in perceived taste intensity. That will have to be tested in future psychophysical experiments in which taste is serving as a cue rather than a reinforcer in a signal detection task. Moreover, we also cannot dismiss the possibility that the effects of the neural isolation of the olfactory bulbs on motivated responses to preferred versus avoided taste compounds interact, in part, with the physiological state promoting stimulus sampling. For preferred stimuli, the internal state was established with food restriction, and for avoided stimuli it was effectuated via water restriction. The resolution of these issues is important to provide functional meaning to any downstream neural repercussions of the bulbotomy.

Another way to view the mechanism of action underlying the effects of bulbotomy on taste function is from the neural vantage point. Of course, this is complementary to and follows from an understanding of the psychophysical effects of the manipulation. The outcomes reported here could potentially be due to a loss of explicit stimulation by odorants (i.e., anosmia) or a loss of a major source of projections to (or from) central sites. With regard to the latter, it is possible that some level of tonic activity, provided from the olfactory and/or vomeronasal systems, amplifies brain circuits involved with processing taste-related positive affect. Alternatively, the removal of key projections from the olfactory bulb could lead to degenerative or reorganizational events in downstream targets. These are not mutually exclusive possibilities. In terms of the effects of anosmia on taste, humans certainly are able to detect and discriminate the taste of sucrose from that of other taste compounds when they are wearing nose plugs, a standard procedure in human psychophysical taste testing, which precludes stimulation of the olfactory system by odorants (Stinton et al., 2010). There is also evidence that humans can detect orally sampled maltodextrin stimuli without the benefit of explicit olfactory stimulation (Lapis et al., 2014, 2016). One way to assess this in rats and mice would be to eliminate the signal more peripherally arising from the olfactory mucosa, as is done with zinc sulfate. As noted above, in rodent models, the effects of zinc sulfate irrigation of the nasal mucosa on preference for taste stimuli have not been compelling in magnitude or consistency. One methodological limitation associated with this treatment is that it is temporary, lasting only a few days, and thus behavioral testing needs to be completed within the time span of its effectiveness (Alberts, 1974; Slotnick and Gutman, 1977). Contemporary genetic silencing techniques offer promise in this regard.

Regardless of whether it is the loss of explicit odorant stimulation or the removal of anatomic connections involving the main and accessory olfactory bulbs, the central sites that fall victim to the neural transection expressly with respect to the maintenance of taste-motivated licking as described here remain to be experimentally revealed. Of course, the function of the third-order neurons of the primary olfactory and vomeronasal systems would all likely be affected (Shipley and Ennis, 1996; Wilson et al., 2015). Areas that receive convergent input from the gustatory and olfactory systems such as the piriform and insular cortices would be candidate sites of action underlying the behavioral effects of bulbotomy on taste function (Van Buskirk and Erickson, 1977; Escanilla et al., 2015; Maier et al., 2015; Maier, 2017; Samuelsen and Fontanini, 2017). It is also quite possible, if not likely, that the indirect loss of input to areas of the brain known to be involved in taste-related affect and reward, such as the basolateral and central nuclei of the amygdala, the striatum, the ventral pallidum, and the ventral tegmental area, leads to the specific effects of bulbotomy on taste-motivated licking.

Answers to the important questions posed above await further experimental scrutiny. In closing, it is worth emphasizing that we are not claiming that taste input generated by the preferred compounds tested here is unnecessary to maintain concentration-dependent licking responses, as the blunted responsiveness to sucrose displayed by the T1R2+T1R3 KO mice clearly attests. Rather, we are saying that it is not sufficient. Our behavioral results clearly demonstrate that manipulations of the olfactory system can profoundly impact certain aspects of responsiveness to taste compounds in the mouse model and highlight the importance of understanding taste function in a broader context that involves the contribution of multiple chemosensory systems.

## Synthesis

Reviewing Editor: Zoltan Nusser, Institute of Experimental Medicine Decisions are customarily a result of the Reviewing Editor and the peer reviewers coming together and discussing their recommendations until a consensus is reached. When revisions are invited, a fact-based synthesis statement explaining their decision and outlining what is needed to prepare a revision will be listed below. Note: If this manuscript was transferred from JNeurosci and a decision was made to accept the manuscript without peer review, a brief statement to this effect will instead be what is listed below.

The current figures are very simple; usually have few panels. The authors should combine figures to reduce the total number of figures. Presenting all statistical data in separate tables is very unusual. I agree with the authors that it disrupts the text and they should not include them in the main text, but the figure legends should be the perfect place for them.
